# Airway Hyperresponsiveness and Quality of Life in Western Red Cedar Asthmatics Removed from Exposure

**DOI:** 10.1371/journal.pone.0050774

**Published:** 2012-12-04

**Authors:** Jian-Qing He, Moira Chan-Yeung, Chris Carlsten

**Affiliations:** 1 Department of Respiratory Medicine. West China Hospital, West China Medical School, Sichuan University, Chengdu, China; 2 Respiratory Division, Department of Medicine, University of British Columbia, and Centre for Lung Health, Vancouver General Hospital, Vancouver, British Columbia, Canada; National Jewish Health, United States of America

## Abstract

**Objectives:**

Most western red cedar asthmatics (WRCA) continue to have symptoms even after removal from exposure. Consequently, health-related quality of life (HRQL) is often impaired. The objective of this study was to evaluate the relationship between two measures of AHR and HRQL scores in those with WRCA.

**Methods:**

HRQL was determined by the short form 36 (SF-36) in 46 male, non-smoking individuals with WRCA removed from exposure to western red cedar, on average, 15 years earlier. The relationships between the SF-36 total score and its eight domains with 2 indices from methacholine-stimulated airway hyperresponsiveness (the provocative concentration of methacholine causing a 20% fall in FEV_1_ [PC_20_] and bronchial reactivity index [BRI]) were analyzed by the Pearson correlation and multiple linear regression.

**Results:**

PC_20_ was significantly correlated with the SF-36 total score and its two domains of bodily pain and general health (r = 0.34, 0.40, 0.40, p = 0.023, 0.006, 0.006, respectively). BRI was significantly correlated with bodily pain and general health (r = −0.35, −0.42, p = 0.017, 0.004, respectively); correlations remain significant after adjusting for age, ethnicity, years since diagnosis, years since last exposure and use of inhaled corticosteroid. BRI and other measures of airway responsiveness were not associated with inhaled corticosteroids use.

**Conclusions:**

In Western red cedar asthmatics removed from exposure, measures of airway responsiveness are associated with HRQL.

## Introduction

Asthma is a chronic inflammatory pulmonary disorder characterized by reversible airflow obstruction caused by a multiplicity of stimuli [1]. About 10% of adult asthmatics have occupational asthma [2]. Western red cedar asthma (WRCA) is the most common type of occupational asthma in the southwestern Canada and in Pacific Northwest of the United States. Between 4 and 13.5% of those working with western red cedar (WRC) in an occupational setting develop asthma via sensitization to plicatic acid, a low molecular weight compound present only in WRC [3]. The majority of subjects with WRCA continue to have respiratory symptoms and live with permanent disability even years after cessation of exposure [4]. Therefore, ongoing treatment is needed for some subjects even long after removal from exposure, and the management is often more challenging than non-occupational asthma because cases of occupational asthma typically involve issues of work compensation and employment [5].

In recent years, it has become clear that the goals of asthma management not only include symptomatic control, but also involve maintaining and improving health-related quality of life (HRQL) [6]. Subjects with occupational asthma have more significant decreases in HRQL compared with their counterparts without occupational exposure [7]. HRQL impairment in asthmatics is not strongly related to traditional clinical symptoms of asthma [8]. Thus, one is motivated to better understand what aspects of asthma contribute to ongoing impairments in HRQL so that clinicians may target relevant components in an attempt to improve HRQL.

Airway hyperresponsiveness (AHR) is a cardinal feature of asthma [1], [9], yet the relationship of AHR with HRQL has been inconsistent within previous studies. The inconsistency may arise from the fact that most of those studies used PC_20_ [the provocative concentration of a substance (usually methacholine) causing a 20% fall in FEV_1_] or PD_20_ [the administered dose of a substance in the inhaled aerosol (usually methacholine) which causes the FEV_1_ to fall by 20%] to represent sensitivity or threshold of AHR. Only two studies used bronchial reactivity as determined by the slope of the dose-response curve, which may better represent the intensity of the bronchoconstriction and the severity of AHR [10], [11]. Porsbjerg and colleagues reported that the presence of AHR and the severity of asthma each contribute independently to quality of life even though the most severe asthmatics had greater prevalence of AHR [11]. More recently, Cisneros and colleagues reported that a bronchial reactivity index was more informative than traditional measures of AHR, such as the PC_20_, in predicting the HRQL of patients with stable asthma; they therefore recommended the use of bronchial reactivity indices to represent AHR [10].

The objective of this study was to evaluate the relationship between two measures of AHR and HRQL scores in those with WRCA. We were motivated particularly to understand the relationship between AHR and HRQL in occupational asthmatics who have been removed from exposure, as this has significant implications for the long term management of such individuals and for the compensation systems that take responsibility for their care. Some of the results of these studies have been previously reported in the form of an abstract [12].

## Methods

### Ethics Statement

The study was approved by the institutional review board of the vancouver coastal health research institute (project approval number: H08-01306). Writing informed consent was obtained from all patients.

### Study Participants

This is a cross-sectional study of subjects diagnosed with WRCA by standardized plicatic acid challenge test at a provincial referral centre at Vancouver General Hospital since 1972. A total of 47 lower mainland residents of British Columbia who were diagnosed with WRCA were eligible and agreed to participate. Methacholine challenge testing could not be successfully performed for one subject; therefore 46 subjects finished the study. The ATS asthma impairment score was assessed and impairment class determined based on post-bronchodilator FEV_1_, AHR and minimum medication needed [13]. According to the validated schema, the class of impairment was expressed as class 0, 1, 2, 3, and 4 (total asthma severity score 0, 1–3, 4–6, 7–9 and 10–11).

### Health-related Quality of Life Scores

All interviews were performed in person by the study coordinator. HRQL scores were assessed with the Short Form 36 (SF-36) [14], one of the most commonly used and validated generic health status questionnaires in adults. Total score and eight domains of HRQL were quantified: physical functioning, physical health, emotional problem, vitality (energy/fatigue), emotional well-being, social functioning, bodily pain, and general health perception. A higher asthma HRQL score reflects better functioning. Values close to 100 indicate excellent overall health and functioning.

### Methacholine Challenge Test

A methacholine challenge test was performed according to the method of Cockcroft et al [15] and the provocative concentration of methacholine that induces a 20% fall in FEV_1_ (PC_20_) was calculated from the following standard formula [16]:
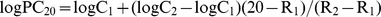
where C_1_ = second-to-last concentration, R_1_ = second-to-last response, C_2_ = last concentration, and R_2_ = last response. In cases where the FEV_1_ dropped between 15% and 20%, the same formula was applied [17], [18]. In cases where the FEV_1_ didn’t drop 15% by the last concentration of methacholine, the last concentration of methacholine was assigned as PC_20_ [17], [18]. The bronchial responsiveness index (BRI) was determined using the method described by Burrows *et *al [19]. The percentage decline in FEV_1_ was divided by the log of the last concentration of methacholine and then 10 was added to the resultant value to eliminate negative values, before log-transformation. FEV_1_% decline was defined as the decline in FEV_1_ from the post-diluent baseline value after the final methacholine was administrated, and the concentration of final dose was defined as the concentration of methacholine in mg/dl.

### Statistical Analysis

The relationships between the HRQL total score and scores from its eight domains with methacholine-stimulated airway hyperresponsiveness (PC_20_ and BRI) were analyzed by Pearson correlation. Then multiple linear regressions were performed to adjust for age, ethnicity, years since diagnosis, years since last exposure and inhaled corticosteroid usage. P-values of ≤ 0.05 were considered significant and p-values of 0.05 to 0.10 were considered of borderline significance. All tests were performed using the JMP5.0 Statistics software package (SAS Institute Inc., Cary, NC).

## Results

### Participant Characteristics

A total of 46 WRCA patients were studied, which accounted for 32% of those subjects that participated in our previous study [20]. When we compared demographic characteristics of participants in the current study with those who didn’t participant in the current study but participated our previous study, there were no significant difference in key demographic characteristics such as age, race, atopy, years from initial diagnosis, or years from last exposure. There was also no difference in scores of health-related quality of life in patients of the two studies.

All 46 participants were male, non-smokers, with an age (mean±SD) of 60.5±10.9, and 60.9% were non-Caucasian. WRCA was diagnosed 24.9±7.2 years previously and removal from WRC exposure occurred 14.8±10.3 years previously. All participants were asthmatics with impairment class of 0 to 2, two patients presenting as class 0, 23 patients presenting as class 1, and 21 patients presenting as class 2. In addition, 41.3% patients were using inhaled corticosteroids (ICS; Table 1).

**Table 1:** Demographic data of participants, their lung function and methacholine challenge test results (n = 46).

Demographic variable	Mean (range) or n (%) or Mean ± SD
Age, years	60.5 (25–82)
Race, non-white	28 (60.9%)
Atopy	16 (36.8%)
Years from initial diagnosis	24.9±7.2
Years from last exposure	14.8±10.3
Asthma score	2.9 (0–11)
Impairment class:	
Class 0	2 (4.3%)
Class 1	23 (50.0%)
Class 2	21 (45.7%)
Inhaled steroid	19 (41.3%)
FEV_1_ (% predicted)	76.18±16.15
FEV_1_/FVC	0.72±0.07
PC_20_ (mg/ml)	6.34±6.55
BRI	1.26±0.14

### Airway Hyperresponsiveness Indices

Bronchial challenge was positive (PC_20_<8.0 mg/dl) for 29 subjects (63.0%) and negative for 17 subjects (37.0%). PC_20_ (mean±SD) (range) was 6.34±6.55 mg/ml (0.13–16.0 mg/ml). Airway reactivity index BRI (mean±SD) (range) was 1.26±0.14 (1–1.54) (Table 1). The relationships between continuous clinical parameters (asthma scores, FEV_1_% predicted and FEV_1_/FVC) and each AHR index was statistically significant (Table 2). Since FEV_1_% predicted is one the three components of asthma severity scores, FEV_1_% predicted is highly correlated with asthma severity score with r = −0.670 and p<0.0001. Years since diagnosis was also significantly correlated with BRI (Table 2). WRCA patients with class 2 impairment had significantly more prominent AHR (lower values of PC_20_) than patients with class 0 and class 1 impairment (data not shown). Race and atopic status were each not correlated with AHR indices (data not shown). Steroid usage was also not correlated with AHR indices (see further detail, in “Association of inhaled corticosteroid usage” section, below).

**Table 2:** Pearson correlation coefficients between the airway responsiveness indices and clinical and spirometric parameters [r (P value)].

Clinical and spirometric parameters	PC_20_, mg/ml	BRI
Age, yrs	−0.04 (0.777)	−0.05 (0.719)
Yrs since diagnosis	−0.180 (0.286)	0.239 (0.146)
Yrs since last exposure	−0.025 (0.868)	−0.099 (0.511)
Score	−0.567 (<0.0001)	0.530 (0.0002)
FEV_1_, % pred	0.530 (<0.0001)	−0.557 (<0.0001)
FEV_1_/FVC	0.397 (0.006)	−0.411 (0.005)

### Health-related Quality of Life Scores and their Correlation with Clinical and Spirometric Parameters

The mean scores for each of the eight SF-36 domains are shown in Table 3; their ranges are 39.1 to 76.9 for the 46 subjects. In comparison with normative data for the general Canadian population [21], [22], the SF-36 scores of the WRCA subjects were significantly lower for all SF-36 domains except bodily pain. The relationships of continuous clinical parameters with the SF-36 scores showed a statistically significant correlation of asthma scores and FEV_1_% predicted with each of physical functioning, general health and SF-36 total score. Asthma scores were also correlated with bodily pain (Table 4). Non-Caucasian WRCA patients had significantly lower scores than Caucasians in each of physical functioning, emotional well-being domains and SF-36 total score (p = 0.017, 0.039 and 0.029 respectively). WRCA patients with class 2 impairment had lower score in general health than patients with class 0 and class 1 impairments (p = 0.034). However, there were no significant differences for all other seven SF-36 functional domains between class 2 with class 1 and class 0 impairments. In addition, steroid usage was significantly correlated with physical functioning score (p<0.0001) but not correlated with scores of other domains and total scores of SF-36 (see further detail, in “Association of inhaled corticosteroid usage” section, below). Atopic status was not correlated with HRQL scores as determined by SF-36 (data not shown). The number of years removal from WRC exposure was also not associated with HRQL scores (data not shown).

**Table 3:** Health-related quality of life scores.*

Functional domain	WRCA (n = 46)	Canadian General Population (N = 9367–9411)	P value
Physical functioning	76.9±24.0	85.8±20.0	0.003
Physical health	47.3±46.3	82.1±33.2	<0.0001
Emotional problem	50.0±48.6	84.0±31.7	<0.0001
Energy/fatigue	47.3±14.6	65.8±18.0	<0.0001
Emotional well-being	39.1±7.7	77.5±15.3	<0.0001
Social functioning	58.2±6.1	86.2±19.8	<0.0001
Bodily pain	73.1±27.5	75.6±23.0	0.463
General health	62.3±8.4	77.0±17.7	<0.0001

Note: *The values are given as the mean±the standard deviation.

**Table 4:** Pearson correlation coefficients between SF-36 scores and clinical and spirometric parameters [r (P value)].

	Age, yrs	Yrs since diagnosis	Yrs since last exposure	Severity score	FEV_1_, % pred	FEV_1_/FVC	PC_20_	BRI
Physical functioning	0.324(0.831)	−0.240 (0.152)	−0.174 (0.246)	−**0.318 (0.031)**	**0.367 (0.012)**	−0.091 (0.548)	0.265 (0.086)	−0.269 (0.070)
Physical health	0.004 (0.980)	−0.194 (0.249)	−0.082 (0.590)	−0.099 (0.515)	0.183 (0.225)	**0.296 (0.046)**	0.209 (0.164)	−0.176 (0.242)
Emotional problem	0.106 (0.485)	−0.042 (0.805)	−0.103 (0.495)	−0.197 (0.190)	0.272 (0.067)	0.182 (0.223)	0.227 (0.129)	−0.175 (0.243)
Energy/fatigue	−0.171 (0.256)	−0.265 (0.133)	−0.102 (0.501)	−0.121 (0.423)	0.209 (0.163)	−0.100 (0.511)	−0.013 (0.930)	0.020 (0.894)
Emotional well-being	−0.134 (0.345)	−0.018 (0.915)	0.072 (0.633)	−0.018 (0.908)	0.022 (0.885)	0.160 (0.289)	0.073 (0.628)	0.007 (0.961)
Social functioning	−0.289 (0.052)	0.271 (0.105)	−0.233 (0.119)	0.124 (0.410)	−0.085 (0.576)	−**0.354 (0.016)**	−0.152 (0.309)	0.236 (0.115)
Bodily pain	−0.064 (0.671)	0.033 (0.847)	−0.151 (0.318)	−**0.379 (0.009)**	0.209 (0.163)	**0.312 (0.035)**	**0.398 (0.006)**	−**0.351 (0.017)**
General health	−0.177(0.240)	−0.001 (0.996)	0.072 (0.633)	−**0.464 (0.001)**	**0.318 (0.031)**	**0.354 (0.016)**	**0.400 (0.006)**	−**0.420 (0.004)**
SF 36 total score	−0.020 (0.894)	−0.147 (0.387)	−0.156 (0.301)	−**0.308 (0.038)**	**0.344 (0.019)**	0.244 (0.102)	**0.335 (0.023)**	−0.285 (0.055)

### Correlations of Airway Hyperresponsiveness Indices with HRQL Scores by SF-36

**Figure 1:**
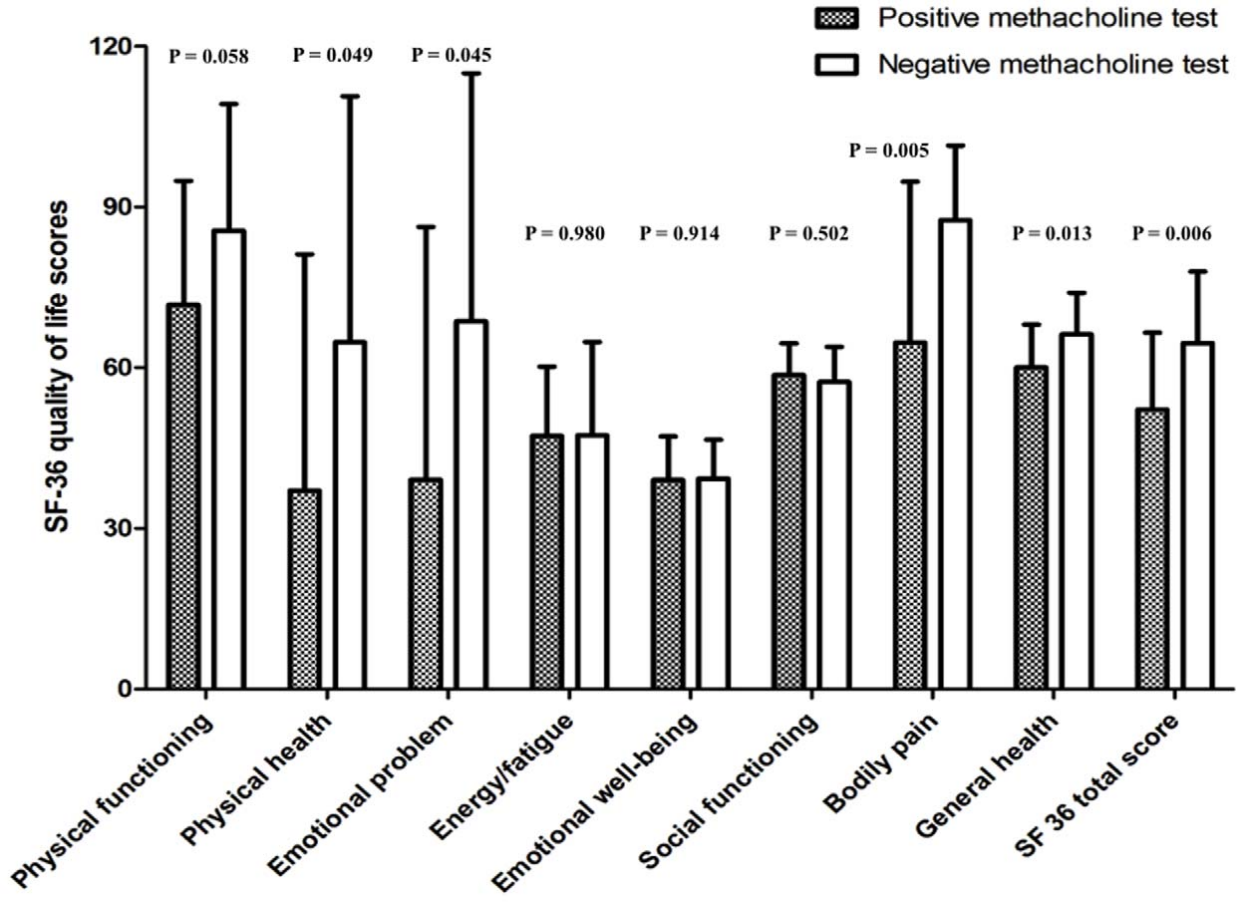
Comparison of SF-36 health-related quality of life scores according to the response to methacholine challenge test.

When the SF-36 scores were assessed in relation to the conventional measure of response to methacholine (PC_20_), the patients with positive methacholine test (PC_20_<8 mg/ml) had lower total SF-36 score and lower scores in 4 of its 8 domains (Figure 1). PC_20_ was significantly correlated with the SF-36 total score and its domains of bodily pain and general health (r = 0.34, 0.40, 0.40 and p = 0.023, 0.006, 0.006 respectively). BRI was significantly correlated with bodily pain and general health (r = −0.35, −0.42; p = 0.017, 0.004, respectively). These correlations are still significant after adjusting for age, ethnicity, years since diagnosis, years since last exposure and inhaled corticosteroid usage.

### Association of Inhaled Corticosteroid Usage with Clinical Parameters, Airway Hyperresponsiveness Indices and HRQL Scores

To test if inhaled corticosteroid usage will reduce AHR or improve quality of life, clinical parameters, AHR indices and HRQL scores were compared (Table 5). In clinical parameters, asthmatics on ICS have higher severity scores than asthmatics not on inhaled corticosteroid (p = 0.013); there is no difference in other clinical parameters such as age, years from initial diagnosis, years from last exposure. In AHR indices, asthmatics on inhaled corticosteroid have lower PC_20_ than asthmatics not on inhaled corticosteroid (4.8 mg/ml versus 7.4mg/ml) but the difference is not statistically different (p = 0.194). In HRQL scores by the SF-36, asthmatics on ICS have lower scores in the physical functioning domain than asthmatics not on inhaled corticosteroid (p = 0.0005). Asthmatics on ICS also have lower scores in the SF-36 total score and its emotional problem domain, but in each case the difference is of borderline statistical significance (p = 0.064, p = 0.08, respectively).

**Table 5:** Association of inhaled corticosteroid usage with clinical parameters, airway responsiveness indices and HRQL scores.

	Asthmatics currently on inhaled corticosteroid	Asthmatics currently not on inhaled corticosteroid	P value
	(n = 19)	(n = 27)	
Age, yrs	61.0±9.6	60.1±11.9	0.798
Yrs since diagnosis	25.5±6.2	24.2±8.7	0.612
Yrs since last exposure	14.7±11.1	15.0±9.9	0.194
Score	3.7±1.5	2.4±1.7	**0.013**
PC_20_ (mg/ml)	4.8±6.3	7.4±6.7	0.194
BRI	1.3±0.1	1.2±0.1	0.317
Physical functioning	60.5±28.2	88.3±10.8	**0.0005**
Physical health	46.1±47.3	48.1±46.5	0.882
Emotional problem	35.1±47.8	60.5±47.2	0.080
Energy/fatigue	44.7±9.4	49.1±17.3	0.325
Emotional well-being	39.8±9.1	38.7±6.7	0.633
Social functioning	57.8±9.0	58.4±2.9	0.721
Bodily pain	70.4±29.2	75.0±26.6	0.582
General health	60.5±8.6	63.6±8.1	0.231
SF 36 total score	51.9±16.5	60.2±13.3	0.064

Note: *The values are given as the mean ± the standard deviation.

## Discussion

The main finding of this cross-sectional study is that AHR is significantly correlated with HRQL in occupational asthmatics even several years after removal from the specific allergen responsible for the onset of their asthma. AHR, qualitatively expressed by positive or negative methacholine test (PC_20_<8 mg/ml or ≥ 8 mg/ml), is associated with total HRQL scores and four of its eight domains. AHR, quantitatively expressed both by bronchial sensitivity (PC_20_), and by bronchial reactivity (BRI), is correlated with HRQL scores in those recovering from WRCA. In addition, AHR was also correlated with asthma severity scores, FEV_1_% predicted, and FEV_1_/FVC. Furthermore, HRQL scores from all eight domains of SF-36 except bodily pain were significantly lower in this group of WRCA patients who has been removed from exposure for an average of 15 years, relative to the reported population norms. Finally, although WRCA patients on ICS had more severe asthma compared with those not on ICS, there was no difference in specific AHR indices.

A recent study Cisneros *et al*. suggested the use of bronchial reactivity indices to express AHR since the bronchial reactivity indices such as BRI were correlated better with the HRQL scores than bronchial sensitivity indices such as PD_20_ [10]. We included bronchial reactivity BRI in our analysis because such a continuous measure also has the advantage of yielding an estimate of airway responsiveness in all subjects, not just those with airway hyperresponsiveness [23]. Although Cisneros *et al* also investigated other indices of airway reactivity (continuous indices of responsiveness and dose-response slope) [10], they are somewhat redundant in that each is calculated from the slope of FEV_1_ drop and methacholine dose. Since Burrows *et al* have shown advantages of BRI relative to other measures of dose-responsiveness, and because articles using BRI to represent airway reactivity indices have been widely published and well-accepted [16], [23], [24], [25], we focused on BRI.

In our study with recovering WRCA, however, both bronchial sensitivity and bronchial reactivity were significantly correlated with HRQL scores. There are several possible in which our results may not be generalizable to all asthmatics. First, patients with WRCA may be different from others with atopic asthma in that airway inflammation persists even long after removing from exposure [26], which may be explained by differences in T-cell subtypes and cytokine profiles compared to that of others with atopic asthma [27]. Second, our subjects are older asthmatics (average age = 60 years) with long disease duration. Studies have showed that older asthmatics have different pathophysiological mechanisms that determine AHR, relative to younger asthmatics [28]. Third, all research subjects in this study are male. In males, AHR may decline with age, while in females, AHR may increase with age [29], [30], [31]. Therefore, participants of this study may have relatively blunted AHR due to their male gender and advanced age. This study confirms previous observations that AHR was associated with asthma severity and that patients with positive methacholine challenge test had lower scores of HRQL [11], [32].

The current study demonstrates that those with Western red cedar asthma, even having been removed from their allergenic exposure for several years, have HRQL scores that are similar to those with allergen-stimulated asthma and lower than that reported of healthy Canadians [33]. As shown in our previous publication on HRQL in WRCA subjects, HRQL scores in WRCA were low in most domains of HRQL scores evaluated by SF-36 [20]. Bodily pain was the only domain that had similar scores between WRCA asthmatics and those reportedly as healthy in various Western populations [34], [35], [36]. One of the possible reasons for this finding is that WRCA asthmatics may not be very sensitive to bodily pain due to their work habits.

It is worth pointing out that in this study, AHR, FEV_1_% predicted, FEV_1_/FVC and asthma severity score are all correlated with HRQL scores. The association between FEV_1_% predicted or asthma severity with HRQL scores is supported by previous studies [37], [38], [39].

Although WRCA patients on ICS had more severe asthma compared with those not on ICS, there was no difference in specific AHR indices, suggesting that corticosteroid-attributable differences in severity were not due simply to differences in AHR. One may hypothesize that the corticosteroids are normalizing AHR in individuals whose ongoing symptom severity is driven by pathophysiology independent of AHR, though exploring this hypothesis in detail was not the intention of this study.

The significance of this study lies in establishing effective, individualized asthma management strategies for WRCA patients removed from exposure. The final goal of asthma management is improving or maintaining quality of life of asthmatics. In this study we concluded that AHR is associated with HRQL scores in WRCA subjects who had left exposure in average 15 years. Therefore, the management strategy must be aimed at reducing AHR. Comparing with our previous study of WRCA removing from exposure for about 5 years, the current group of patients had reduced asthma impairment class and less percentage of steroid usage, yet two-third of WRCA subjects in this group still experience positive methacholine challenge test and reduced HRQL scores. Therefore for WRCA patients, even long removed from exposure, reducing AHR remains fundamental to management. Given the known effectiveness of ICS for asthma in general, we recommend in this population especially those have AHR that ICS continue to be used according to standard asthma management guidelines until such time that a prospective study may demonstrate that removal of such treatment is not detrimental.

There are several limitations of this study. First, the participation rate was lower than anticipated and therefore the chance of selection bias is considerable. However, subject characteristics were similar to those of a large prior study. A second limitation is that the study is cross-sectional; longitudinal changes and their correlations between AHR and HRQL could not be assessed.

In summary, we found that measures of airway responsiveness, both in terms of bronchial sensitivity and bronchial reactivity, are associated with HRQL in occupational asthmatics even years after removal from exposure. We conclude that AHR is associated with decreased quality of life in occupational asthmatics long after exposure cessation. Therefore, the prevention strategy needs to be strongly aimed at reducing AHR. The primary preventive strategy remains minimizing exposure, exposure intensity, and exposure duration. Once distant from exposure, however, it is naïve to expect that time alone will ‘cure’ the disease in those that have it. Comparing with our previous study of WRCA removing from exposure for approximately 5 years (on average), the group in the present study had improvements in asthma impairment including less inhaled steroid usage, yet two-thirds of those in this group still have a positive methacholine challenge test and HRQL scores that are reduced to a clinically significant degree. Thus, the secondary significance of this study lies in establishing effective, individualized asthma management strategies for WRCA patients removed from exposure. For many WRCA patients, even long removed from exposure, reducing AHR by appropriate pharmacologic agents must remain central to management. Noting that use of ICS appears to diminish over time, in spite of ongoing AHR, care providers should resist this trend and compensation systems should continue to support payment for such medications so long as AHR persists.
